# Evidence of a genetic background predisposing to complex regional pain syndrome type 1

**DOI:** 10.1136/jmg-2023-109236

**Published:** 2023-10-10

**Authors:** Samiha S Shaikh, Andreas Goebel, Michael C Lee, Michael S Nahorski, Nicholas Shenker, Yunisa Pamela, Ichrak Drissi, Christopher Brown, Gillian Ison, Maliha F Shaikh, Anoop Kuttikat, William A Woods, Abhishek Dixit, Kaitlin Stouffer, Murray CH Clarke, David K Menon, C Geoffrey Woods

**Affiliations:** 1 Medical Genetics, Cambridge Institute for Medical Research, Cambridge, Cambridgeshire, UK; 2 Pain Research Institute, Clinical Sciences Centre, University of Liverpool Faculty of Health and Life Sciences, Liverpool, UK; 3 Department of Medicine, Addenbrooke's Hospital, Cambridge, Cambridgeshire, UK; 4 Department of Rheumatology, Addenbrooke's Hospital Rheumatology Department, Cambridge, Cambridgeshire, UK; 5 Department of Biomedical Sciences, Universitas Padjadjaran, Bandung, Indonesia; 6 Heart and Lung Research Institute, Cambridge Biomedical Campus, Cambridge, Cambridgeshire, UK; 7 Brain Physics Laboratory, University of Cambridge Department of Clinical Neurosciences, Cambridge, Cambridgeshire, UK

**Keywords:** Genetic Predisposition to Disease, Genetics, Medical, Genetic Variation, Human Genetics, Mutation, Missense

## Abstract

**Background:**

Complex regional pain syndrome type 1 (CRPS-1) is a rare, disabling and sometimes chronic disorder usually arising after a trauma. This exploratory study examined whether patients with chronic CRPS-1 have a different genetic profile compared with those who do not have the condition.

**Methods:**

Exome sequencing was performed to seek altered non-synonymous SNP allele frequencies in a discovery cohort of well-characterised patients with chronic CRPS-1 (n*=*34) compared with population databases. Identified SNP alleles were confirmed by Sanger sequencing and sought in a replication cohort (n*=*50). Gene expression of peripheral blood macrophages was assessed.

**Results:**

In the discovery cohort, the rare allele frequencies of four non-synonymous SNPs were statistically increased. The replication cohort confirmed this finding. In a chronic pain cohort, these alleles were not overexpressed. In total, 25 out of 84 (29.8%) patients with CRPS-1 expressed a rare allele. The SNPs were rs41289586 in *ANO10*, rs28360457 in *P2RX7*, rs1126930 in *PRKAG1* and rs80308281 in *SLC12A9*. Males were more likely than females to have a rare SNP allele, 8 out of 14 (57.1%) vs 17 out of 70 (24.3%) (Fisher’s p=0.023). *ANO10*, *P2RX7*, *PRKAG1* and *SLC12A9* were all expressed in macrophages from healthy human controls.

**Conclusion:**

A single SNP in each of the genes *ANO10, P2RX7, PRKAG1* and *SLC12A9* was associated with developing chronic CRPS-1, with more males than females expressing these rare alleles. Our work suggests the possibility that a permissive genetic background is an important factor in the development of CRPS-1.

**What is already known on this topic**

- Complex regional pain syndrome type 1 (CRPS-1) is a rare, poorly treatable, chronic pain phenotype arising after injury in some people; its aetiology is unknown.

**What this study adds**

- The discovery of a genetic background composed of four SNPs permissive for the development of CRPS-1, in about a third of the cases.
- While CRPS-1 is less common in males, they were more likely to have genetic findings, suggesting that CRPS-1 has sex-specific aetiological causes.

**How this study might affect research, practice or policy**

- This study provides evidence that some people develop CRPS-1 due to genetically altered disease susceptibility.
- Further study of these genes and SNPs may catalyse the generation of personalised precision diagnosis and treatments for CRPS-1.

## Introduction

Complex regional pain syndrome 1 (CRPS-1; Orphanet reference 99995) is a debilitating pain disorder predominantly affecting a distal limb. In 95% of cases, it occurs post-traumatically and involves sensory, motor and autonomic systems, as well as trophic and bone changes in some patients.^1–4^ There is good evidence to suggest that, at least in the first year of CRPS, local inflammation continues to be present in the affected limb. The affected limb is often warm, red and oedematous in early CRPS within 6 months of injury.^5 6^ Blister fluid from the skin of the affected limb has high levels of the proinflammatory cytokines interleukin-6 (IL-6) and tumor necrosis factor-α (TNF-α) when compared with the contralateral limb’s skin blister.^7^ Early anti-inflammatory treatment with glucocorticoid in patients with CRPS can be effective.^8^ It is difficult to explain the peripheral tissue changes in skin and bone in particular through a solely neurogenic mechanism.

It is uncertain why some individuals develop CRPS and others do not after the same trauma. A heritable component to CRPS has been suggested^9^ and leads to the hypothesis that there is a genetic predisposition to developing CRPS-1 following trauma. Such ‘predisposition’ genes are well recognised in other phenotypes and become physiologically relevant only when exposed to the environmental trigger. For example, aminoglycoside induced deafness is much more likely to occur when a person carries the rare alleles of either SNP rs267606617 or rs267606618,^10^ and toxic epidermal necrolysis after carbamazepine use almost exclusively occurs in carriers of the rare allele of SNP rs3909184.^11^ Therefore, we sought evidence for a genetic background by seeking altered SNP allele frequencies in well-defined cohorts of patients with CRPS-1 compared with population control data.

## Methods

### Case cohorts

The CRPS-UK Registry is a well-characterised cohort of patients with CRPS meeting the Budapest clinical criteria.^3^ Briefly, patients had to show symptoms in three domains and signs in two domains of the following categories: sensory, vasomotor, sudomotor, motor/trophic. Participants from the CRPS-UK Registry were approached to form a discovery cohort (n=34). A replication cohort was also recruited from the CRPS-UK Registry with the addition of patients’ blood samples stored from the LIPS study.^12^

This resulted in a combined total of 84 patients with CRPS-1 who were able to provide DNA for the study; 34 as a discovery cohort, and then as we had potentially interesting results, 50 as a replication cohort. For our chronic pain cohort (CPC) (n=39), we had recruited patients with fibromyalgia (American College of Rheumatology (ACR) 2016 criteria) and low back pain sufficiently severe that it required an implanted spinal cord stimulator or residential pain management attendance. They were contemporaneously recruited and geographically co-located. All patients (in all three cohorts) had a minimum pain duration of 12 months.^3 13^ The three patient cohorts are summarised in table 1.

**Table 1:** Demographics of study cohorts

Cohort	CRPS-1 discovery	CRPS-1 replication	Chronic severe pain^1^
Total individuals studied	34	50	39
Age range	51 (23–73)	47 (20–73)	54 (26–86)
Females	28	42	26
Age range	51 (23–73)	47 (23–73)	50 (26–74)
Males	6	8	13
Age range	51 (32–56)	48 (20–66)	61 (38–86)

For each of the three cohorts reported in this study, the number of individuals studied is given, and the age range in years (mean followed in parenthesis by minimum and maximum). Then, the number and age data by sex are given for each cohort. All study individuals were Caucasian, but this was not because of the exclusion of other ethnicities.

Individuals with chronic severe back pain untreatable by analgesics; there were no diagnoses of CRPS and neuropathic pain. All individuals had either a spinal cord stimulator implant or attended the Residential Guys and St Thomas’s residential INPUT pain management course.

CRPS-1, complex regional pain syndrome type 1.

All patients gave informed consent. The cohorts of CRPS-1 and chronic pain individuals were collected over a 5-year period starting from 2014 after NHS Health Research Approval for ethical research.

### Exome sequencing and analysis of exome results

DNA was extracted from peripheral blood taken into EDTA tubes for all three cohorts, and assessed for concentration (≥12.5 ng/µL) and integrity (nanodrop spectrophotometer analysis of the 260/280 column range being between 1.80 and 2.00). For the CRPS-1 discovery cohort and the CPC, their genomic DNA was used for next-generation sequencing performed by Beijing Genomics Institute. SureSelect Agilent V4 (51M) kit (Agilent Technologies, Santa Clara, California, USA) was used for exon capture and library preparation. Targets of 100–150 bp were sequenced with an IlluminaHigSeq2000 100PE platform to an average of 50-fold coverage, as previously described.^14 15^ Reads were aligned to the human genome GRCh37/hg19 reference genome (http://genome.ucsc.edu/) by BWA (http://bio-bwa.sourceforge.net/). The SNPs and indels were detected by the variant calling Samtools bcftools program (http://samtools.sourceforge.net/) as this was the best available program at the time fSNPd was written. Such an analysis does not include all parts of all exons of all human genes. The average coverage of exonic bases per individual is between 93% and 97%; however, there is consistency in which exonic regions have poor coverage.^16^

### Analysis of exome sequencing data

**Figure 1:**
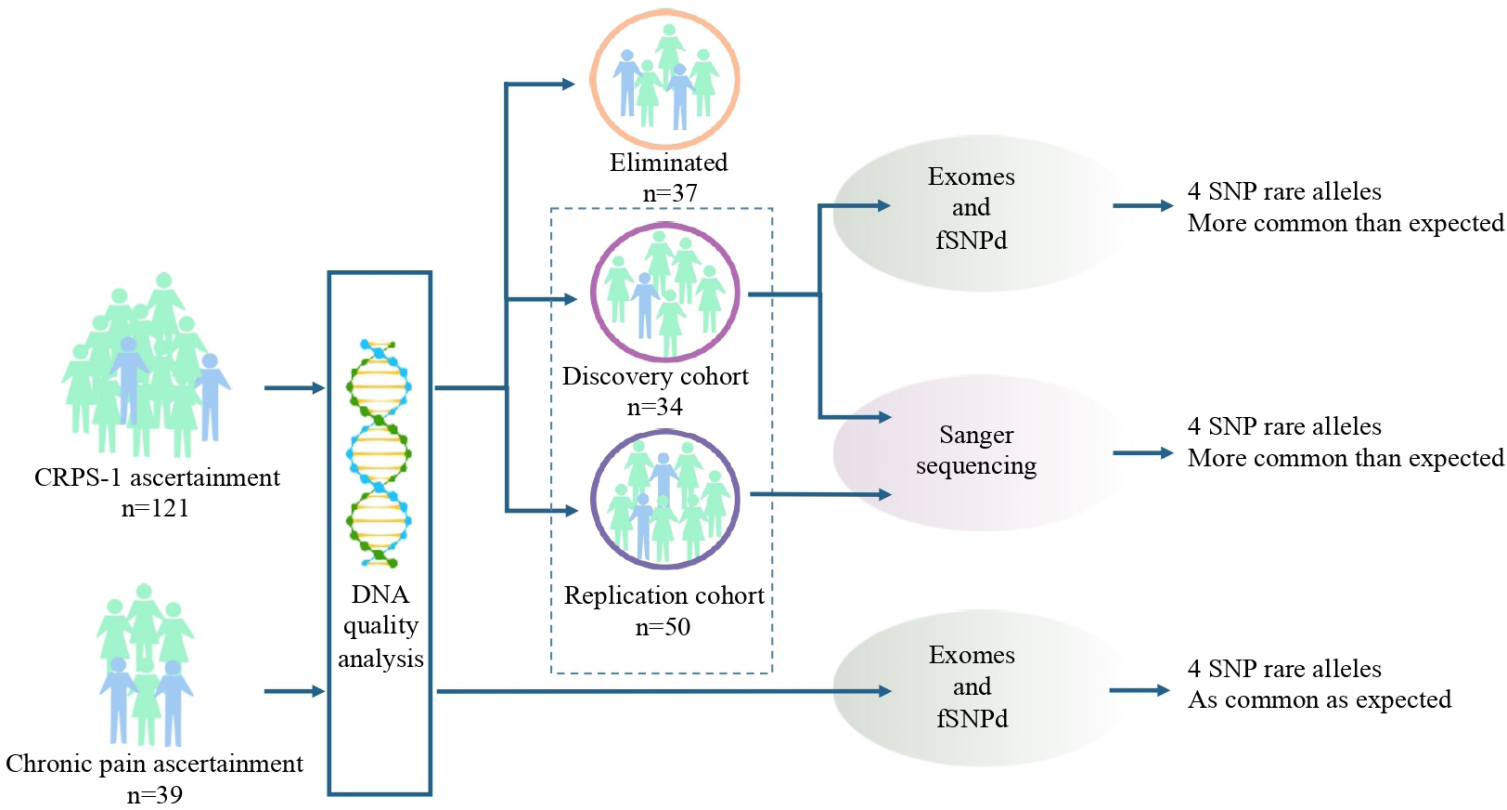
SNP identification through exome sequencing of the discovery and replication cohorts. We had a total of 121 patients with chronic complex regional pain syndrome type 1 (CRPS-1). Of these, 34 individuals formed the discovery cohort, 50 individuals formed the replication cohort and 37 individuals whom we could not use due to insufficient quality of genomic DNA. Our chronic pain cohort consisted of 39 individuals with fibromyalgia and low back pain. Exon capture and next-generation sequencing was performed on genomic DNA from the 34 CRPS-1 individuals of the discovery cohort and the 39 individuals of the chronic pain cohort by Beijing Genomics Institute. The Agilent 51M kit sequenced to an average of 50-fold coverage was performed, as previously described.^14 15^ Such an analysis does not include all parts of all exons of all human genes. The average coverage of exonic bases per individual is between 93% and 97%; however, there is consistency in which exonic regions have poor coverage.^16^ We identified four SNPs where the rare allele was more common than expected in the discovery cohort. However, in the chronic pain cohort, the rare SNP allele was as common as expected. These four SNPs were Sanger sequenced in the replication cohort.

From the individual exome sequencing results, we used the vcf, bam and bam.bai files for exome-wide SNP allele frequency assessment using the fSNPd program.^16^ This identifies the majority of exonic and near-exonic SNPs sequenced within each exome, including the depth and quality of the sequence data, and the alleles detected. For all SNPs, especially those whose rare allele frequency is <5%, geographical and ethnic differences must be considered. Therefore, for each SNP, the allele frequencies were compared with normative values from reference databases which we considered likely to be geographically and ethnically like our cohorts; the 1000 Genome project cohort composed of UK individuals only, and the Exome Variant Server database (northern European). The significance of deviations from expected frequency was assessed using a χ^2^ test with two-tailed and Yates’s correction. The resulting p values were subjected separately to a Bonferroni correction and false discovery rate (FDR) correction, as approximately 100 000 SNPs were detected and assessed in the exome of each individual (see figure 1 and results in table 2 and online supplemental tables 1, 2, 3 and 5).^16 17^

10.1136/jmg-2023-109236.supp1 Supplementary data

**Table 2:** Genetic results for the study cohorts

Gene and SNP	SNP alleles	CRPS discovery cohort allele frequency (n=34)	1000 Genomes χ^2^ p value (FDR corrected)	EVS χ^2^ p value (FDR corrected)	CRPS discovery and replication cohorts allele frequency (n=84)	CRPS combined cohort data uncorrected p value against EVS	CRPS combined cohort data uncorrected p value against UK Biobank	Chronic pain cohort allele frequency (n=39)
*ANO10* rs41289586	p.Arg263His	0.088 (6/68)	1.52×10^–6^	0.018	0.065 (11/168)	0.0016	0.0029	0 (0/39)
*P2RX7* rs28360457	p.Arg307Gln	0.059 (4/68)	1.47×10^–6^	0.04	0.030 (5/166)	0.0869	0.236	0 (0/39)
*PRKAG1* rs1126930	p.Thr57Ser	0.103 (7/68)	7.5×10^–7^	0.05	0.054 (11/168)	0.0267	0.0221	0 (0/39)
*SLC12A9* rs80308281	p.Ile350Thr	0.044 (3/68)	6.91×10^–11^	8.53×10^–5^	0.029 (5/168)	0.0001	0.0001	0 (0/39)

The SNP names with the gene they occur in, and common and rare alleles are shown in the first two columns. In each cohort, ‘*n’* refers to the number of people in a cohort. The study results are given for each SNP rare allele for the discovery CRPS-1 discovery cohort and combined CRPS-1 cohorts, and the chronic pain cohort (CPC). Published SNP allele frequencies for 1000 Genomes GBR cohort (most like our cohorts), the Exome Variant Server (EVS) European American cohort (predominantly northern European) and the UK Biobank are shown in table 3. We include population cohort allele frequencies in a more complete version of table 2 (see online supplemental table 5). Cohort study results are given for each SNP as allele frequency uppermost, and then in brackets are the number of rare alleles over total alleles. For each SNP in the CRPS-1 discovery cohort, the statistical difference of a false discover rate (FDR) corrected (against the total number of SNPs, see text) χ^2^ without Yate’s correction two-tailed p value is given. This is shown against 1000 Genomes reference data and EVS data separately. For the combined CRPS-1 cohort (discovery and replication cohorts) results we conducted a χ^2^ (without Yates correction) two tailed test against EVS and UK Biobank SNP allele frequencies. The final column shows the results of an fSNPd discovery analysis for our chronic pain cohort (CPC) with all four SNPs with genotypes and coverage confirmed; statistics are not given as no individuals of the cohort had any of the CRPS-1 SNP rare alleles.

**Figure 2:**
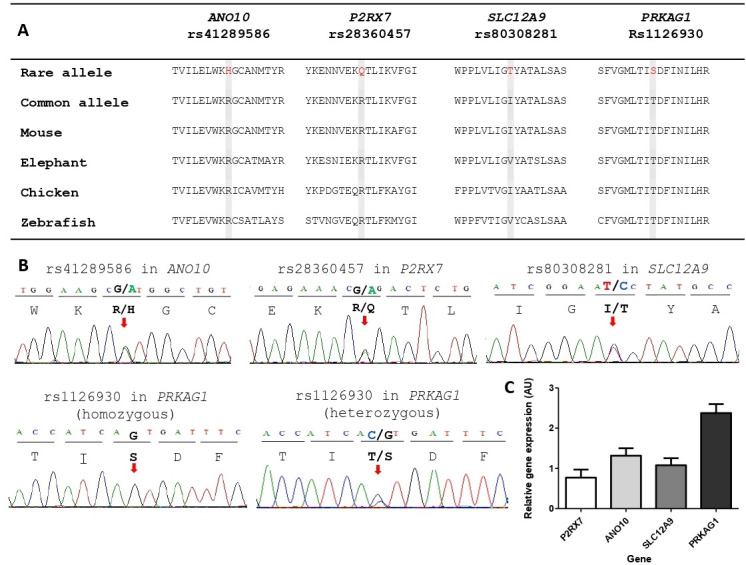
SNP allele amino acid evolutionary conservation, electrophoretograms of each SNP and gene expression of SNP genes in human macrophages. (A) The amino acid each complex regional pain syndrome type 1 (CRPS-1) SNP rare allele encodes is shown in red bold in the first line. The second line shows the wild-type/common amino acid in black. They are shown in the context of their 18 surrounding amino acids. Below the wild-type line are shown the corresponding orthologous peptide sequence from representative vertebrates; mammals (mouse and elephant), birds (chicken) and fish (zebrafish). Data are from the conservation track of the Human Genome Browser. (B) The electrophoretograms of Sanger sequencing of CRPS-1 study individuals. The first three images show heterozygous results for the SNP alleles of rs41289586 in *ANO10*, rs28360457 in *P2RX7* and rs80308281 in *SLC12A9*. The last two images show the result for the homozygous rare allele and heterozygous rare allele of rs1126930 in *PRKAG1*. (C) Peripheral human macrophage expression results, generated by qPCR, for each of the four genes in which we found CRPS-1 associated SNPs. Macrophages were generated from blood monocytes from three healthy individuals and results are of three repeats.

SNPs identified with a potentially significant p value were assessed and filtered by bioinformatics. Starting by only analysing missense, nonsense, start codon, splicing and small INDEL variants, we then sought those SNPs where the alternative allele was predicted to have deleterious effects on the translated protein. For this, we used the resources within the Human Genome Browser and NCBI BLASTP, for identifying potential protein altering changes and protein sequence comparisons of amino acid evolutionary conservation, Conserved Domains (CD search) for detecting if the amino acid change occurred within a known protein domain, and the SIFT and REVEL programs to predict if missense mutations were deleterious (see figure 2 and table 3).^17^

**Table 3:** Details of the four CRPS-1-associated SNPs found in the discovery cohort

SNP details	Gene	*ANO10*	*P2RX7*	*PRKAG1*	*SLC12A9*
	**SNP**	**rs41289586**	**rs28360457**	**rs1126930**	**rs80308281**
Chromosome location hg19		chr3: 43 618 558	chr12: 121 613 129	chr12: 49 399 032	chr7: 100 457 478
Nucleotide change		C>T	G>A	G>C	T>C
Wild type		Arg 263	Arg 307	Thr 89	Ile 350
Rare allele		His	Gln	Ser	Thr
EvoCon*		=Arg	=Arg	=Thr	Ile>>Val
CDD		Pfam 04547 Anoctamin superfamily	Pfam 00864 ATP P2X receptor	Pfam00571 CBS domain	Pfam00324 Amino acid permease domain
1000G allele frequency		0.0096	0.0037	0.009	0.0005
EVS allele frequency		0.026	0.014	0.034	0.003
gnomAD allele frequency		0.0269	0.0148	0.033	0.0038
UK Biobank allele frequency		0.0277	0.0177	0.0336	0.0049
SIFT score		0	0.01	0.04	0.006
REVEL score		0.755	0.398	0.478	0.775

*EvoCon is an abbreviation of evolutionary conservation, and the data taken from the Human Genome Browser (see figure 2). = means complete conservation between species; >> means the first amino acid is generally conserved with the second been rare; > means that the left amino acid is more common than the right amino acid. CDD is the NCBI Conserved Domain Database, which is part of the NCBI BLAST analysis suite. EVS is the Exome Variant Server. gnomAD is the Genome Aggregation Database. SIFT is the ‘Sorting Intolerant From Tolerant’, a program that predicts whether an amino acid substitution affects protein function, where the score 0 is predicted pathogenic and one is predicted harmless. REVEL is an ensemble scoring programme combining pathogenicity predictions from 18 individual scores, where the score 0 is harmless to one which is pathogenic.

CRPS-1, complex regional pain syndrome type 1.

### Sanger sequencing confirmation of SNPs discovered in discovery cohort exome sequencing

The SNPs identified in the discovery cohort were all Sanger sequenced in all members of the discovery cohort using genomic DNA to assess the fidelity of the exome and SNP calling processes (no errors were found) (see figure 2 and online supplemental table 4). Sanger sequencing of the replication and CPC was also performed. Primer3 was used to design all sequencing primers (https://primer3.ut.ee/). A Bio Rad Tetrad 2 4 Bay PCR Thermal Cycler was used for amplification; conditions used and SNP primer sequences are available on request. Sanger sequencing was outsourced to Genewiz, Azenta Life Sciences.

### SNP allele frequency assessment in the CRPS replication, combined CPRS and additional chronic pain cohorts

The SNPs identified in the discovery cohort were then Sanger sequenced in all individuals of the CRPS-1 replication cohort. The results for the CRPS-1 discovery (online supplemental table 1) and replication cohort (online supplemental table 2) were combined (table 2, online supplemental table 5). They were then analysed for statistical significance using the χ^2^ test, using the same two normative data sources as before (1000 Genomes and Exome Variant Server database, see table 2 and online supplemental tables 1, 2, 3 and 5). We assessed our results against two further genetic population databases. The gnomAD database was released after our fSNPD program was written and initial analysis performed, but included far greater numbers of individuals for ‘non-Finnish all Europeans’ (V.3.1.2, n=34 014 individuals of European non-Finnish descent) than 1000 Genomes or EVS (see table 3). However, there was little difference in the allele frequency and the derived statistics from the EVS data, so gnomAD data are not shown. The UK Biobank data, which gives British SNP allele frequency data (without original ethnicity) for up to 488 377 individuals, similarly became available after our preliminary analysis and we have used these data for our combined CRPS-1 cohort analysis (see table 2 and online supplemental table 5). The genetic results of each CRPS-1 cohort were also analysed by sex (see table 4).

**Table 4:** The CRPS-1 combined cohorts SNP allele results analysed by sex

CRPS-1 cohort by sex	Gene	*ANO10*	*P2RX7*	*PRKAG1*	*SLC12A9*	Individual with any SNP rare allele
	**SNP**	**rs41289586**	**rs28360457**	**rs1126930**	**rs80308281**	
	**Alleles**	**Arg234His**	**Arg307Gln**	**Thr89Ser**	**Ile350Thr**	
Female heterozygotes		7/70	3/70	3/70	2/70	17/70*
Female homozygotes		0/70	0/70	3/70	0/70	
Male heterozygotes		4/14	2/14	2/14	3/14	8/14
Total		11/84	5/84	11/84	5/84	25/84†

For each SNP, the number of females and males that were a heterozygote is given; for *ANO10*, *P2RX7* and *SLC12A9*, there were no homozygotes. For *PRKAG1* alone, three study individuals were homozygotes, and all three were female. For each SNP column, the bottom row shows the total number of individuals who were heterozygous or homozygous for the rare allele. The final column shows the number of females and then males who were heterozygous or homozygous for the rare allele, as well as the total for the combined CRPS-1 cohorts.

*‘Any SNP rare allele’ total for females includes both heterozygous and homozygous results.

†Three individuals had two rare allele SNPs, and one had three. So, in total, 25 individuals were heterozygous or homozygous for one or more rare SNP alleles.

CRPS-1, complex regional pain syndrome type 1.

Of note, for *P2RX7*, our SNP rs28360457 always occurred on a haplotype background defined by the rare allele of the SNP rs7958311 (p.Arg270His; European allele frequency=0.25).^18^ However, once the effect of rs28360457 was removed, there was no signal in our CRPS-1 cohort for rs7958311, by fSNPd re-analysis of the CRPS-1 cohort after removing individuals heterozygous for the rare allele of rs28360457.

We also compared the SNP results to those we had generated from our CPC, where CRPS-1 was specifically excluded as a diagnosis, which were analysed as described for the CRPS-1 discovery cohort (see table 2, online supplemental tables 3 and 5).

### Macrophage expression studies of *ANO10*, *P2RX7*, *PRKAG1* and *SLC12A9*

Human peripheral blood mononuclear cells were isolated from blood from three healthy individuals who were well and not in pain, as previously described.^18 19^ Monocytes were purified from peripheral blood mononuclear cells using EasySep Human CD14 Positive Selection Kit II (StemCell Technologies) and were differentiated into macrophages in complete RPMI 1640 supplemented with 100 ng/mL macrophage-colony stimulating factor, as previously described.^19 20^

Total macrophage RNA was harvested using RNeasy plus kit (Qiagen) according to the manufacturer’s protocol and the first strand of cDNA was synthesised using QuantiTect Reverse Transcription Kit (Qiagen). LightCycler 480 SYBR Green Master (Roche) was used to perform qPCR for *ANO10*, *P2RX7*, *PRKAG1* and *SLC12A9* expression. *GAPDH* was used as the endogenous control and to generate a reference level to compare other gene results to. Primer sequences are available on request.

## Results

### Study cohort demographics

The demographics of the discovery (n=34), replication (n=50) and chronic pain control (n=39) cohorts are given in table 1 and show that the mean age (50 years) and age range were very similar between the three cohorts. The sex ratios were almost identical for the two CRPS-1 cohorts: 82% of CRPS-1 individuals were female and 67% of the CPC were female. Our CRPS-1 cohorts’ demographic findings agree with previous European reports.^1 21^

While we did not select or exclude study recruitment based on ethnicity, all of our study individuals were Caucasian.

### Identification of non-synonymous SNPs where the rare allele frequency was significantly different in the CRPS-1 cohorts

In our CRPS-1 discovery cohort of 34 individuals, we identified four non-synonymous SNPs where the rare allele frequency was altered to a statistically significant extent compared with the 1000 Genome project GBR and EVS data (against which the fSNPd program was written). In each case, the rare allele was more common than expected (see table 2, table 3, online supplemental tables 1 and 5). The SNPs were rs41289586 in *ANO10*, rs28360457 in *P2RX7*, rs1126930 in *PRKAG1* and rs80308281 in *SLC12A9* (see table 3). The discovery cohort results were confirmed in all cases by use of Sanger sequencing and no additional rare alleles were found. Next, we assessed the allele frequency of the four SNPs in our CRPS-1 replication cohort, where none of the rare allele frequencies were as large as in the discovery cohort, (see table 2 and online supplemental tables 1,2 and 5). Combining the discovery and replication CRPS-1 cohorts, all four of the SNPs rare alleles were over-represented compared with the four population databases. These results were statistically significant against all four population databases (1000 Genomes, EVS, gnomAD-data not shown and UK Biobank) for the heterozygous *ANO10*, *PRKAG1* and *SLC12A9* results. The heterozygous P2RX7 rare allele frequencies did not reach significance against the EVS and UK Biobank data. Of the four SNPs, 25 out of 84 (29.8%) of the CRPS-1 cohorts had one or more of these (see table 4).

The CRPS-1 cohorts were also analysed by sex (see table 4). Seventeen of the 70 females with CRPS-1 had one or more of the rare SNP alleles, and eight of the 14 males. This male predominant sex difference reached statistical significance (p=0.023, Fisher’s exact test, two-tailed). We found no evidence of an age effect (results not shown).

### Macrophage expression studies of gene expression

While there is widespread distribution of these genes, all four genes are involved in monocyte/macrophage and related lineage cell function. Hence, we looked at the gene expression in macrophages. We found that *ANO10*, *P2RX7*, *PRKAG1* and *SLC12A9* were all expressed in unstimulated human macrophages isolated from three healthy donors (see figure 2). These results agree with previous studies. *ANO10* has widespread expression, including in macrophages, osteoblasts and microglia (both macrophage-like cells).^22^ *P2RX7* has an expression pattern restricted to mast cells, macrophages, osteoclasts and microglia.^23 24^ *PRKAG1* has ubiquitous expression and *SLC12A9* is expressed only in white cell lineages, particularly monocytes and neutrophils.

## Discussion

We found that the rare allele of four SNPs in four genes were more common than expected in individuals who had suffered CRPS-1 for more than a year; rs41289586 in *ANO10*, rs28360457 in *P2RX7*, rs1126930 in *PRKAG1* and rs80308281 in *SLC12A9*. These results reached significance against the UK Biobank data except for those of rs28360457 in *P2RX7*. The allele frequencies of these SNP were not altered in a contemporaneous chronic pain cohort. We did not detect any differences in allele frequencies in other SNPs in the same four genes. If our findings are correct, then this suggests that these four specific SNPs are each able to increase the risk of a person developing CRPS-1 after injury. All of these genes are polymorphic (see dbSNP and gnomAD), and the finding of these four particular SNPs, but not other non-synonymous SNPs in the same genes, may infer that they cause novel changes of function compared with other SNPs.

For these SNPs to predispose a person to develop CRPS-1, they must cause alterations in the function of the proteins ANO10, P2RX7, PRKAG1 and SLC12A9. That affected individuals were heterozygous for a rare SNP allele strongly suggests each is acting as a dominant negative, with exception of PRKAG1 in females, which might have a recessive or a double dominant effect. Looking at the genes identified in this study, ANO10 is a member of the anoctamin protein family which are active in the membrane of the endoplasmic reticulum, and ANO10 causes endoplasmic reticulum Ca^2+^ store release on stimulation of G-protein coupled receptors.^25 26^ P2RX7 is a cell membrane ATP-ligand gated cation channel that has multiple roles in nociception, inflammation and immunity.^24 27^ AMPK is a heterotrimer consisting of an alpha catalytic subunit, and the two regulatory subunits β and γ (which is PRKAG1). PRKAG1 is required for AMPK to detect and respond to the intracellular ATP-ADP-AMP ratio as it is able to bind all three adenine nucleotides^28^ and is critically involved in CSF1 (colony-stimulating factor 1) induction of human monocyte differentiation. SLC12A9 is the most disparate member of a family of electroneutral cell membrane sodium/potassium chloride cotransporters and little is known about its activity or functions, but it is postulated to be a cell membrane Cl^-^/cation cotransporter.^29^

Turning now to the known or potential effects of the four SNPs on protein function. ANO10 rs41289586 alters an evolutionarily invariant Arginine263Histidine and has been found to inhibit macrophage migration through reducing intracellular Ca^2+^ signalling.^23 28^ P2RX7 rs28360457 encodes for Argine307Glutamine and has been reported to cause a loss of P2RX7 ion conduction, to affect the ability of ATP to bind and to act as a dominant negative.^18^ The PRKAG1 SNP rs1126930 encodes for Threonine89Serine, one of the amino acids directly involved in the γ-subunit’s ability to bind AMP, so could alter AMP or ADP or ATP binding.^30–32^ SLC12A9 SNP rs80308281 changes Isoleucine350Threonine which is in the proteins’ eighth transmembrane domain. This is within the predicted Na^+^/K^+^/Cl^-^ binding cotransporter domain, and so could alter cotransporter function.^29^ In summary, for three of the SNPs, there are good grounds to expect that the rare allele would alter protein function.

ANO10, P2RX7 and SLC12A9 are expressed in immune cells, and in particular cluster to monocytes, macrophages and neutrophils as seen in Human Protein Atlas. ANO10, P2RX7 and SLC12A9 are expressed in the peripheral nervous system neurons, as seen in MouseBrain. PRKAG1 has a ubiquitous expression.

It is well known that tissue injury results in rapid early response by neutrophils, followed by macrophages which contribute to the acute phase of inflammation.^33^ This inflammatory response is a normal post-traumatic physiological event, but in CRPS it is exaggerated.^34^ Inflammatory mediators that are secreted are thought to contribute to trophic changes and peripheral sensitisation of neurons. The role of inflammation in the development and propagation of CRPS is not a novel concept and has been well documented.^7 35 36^

The unpicking of the pathophysiology of CRPS will not simplistically align to a single cell lineage, but the alleles identified are plausible clustered to have a biological response that would align with the clinical presentation of sensory, motor and autonomic changes. Macrophage-cell lineage (tissue resident macrophages, microglia, osteoclasts) are well placed to account for many of the changes seen, hence we confirmed that all four genes are expressed in human peripheral macrophages. In addition, recent mouse studies provide evidence that spinal macrophages can be actively anti-inflammatory after superficial injury—our SNPs may block this process.^37^ Or alternatively, as suggested by recent mice studies of the effects of plasma from humans with CRPS, is the inflammation local but the nociceptive signal is at the dorsal root ganglia and mediated by microglia?.^38 39^ Finally, the pathogenesis of CRPS-1 maybe complex, with the SNP alleles we have found (and autoantibodies found by others) acting in multiple cell types, in different cell processes (eg, ANO10 is seen in inhibitory nerves and has an important role in endolysosomal pathways and P2RX7 is seen in excitatory neurons), and in both peripheral and central locations. Carefully performed functional studies in a variety of cell types will be needed to fully understand the role that the rare alleles have in understanding the disease mechanisms of this enigmatic condition.

Analysis of our results by sex produced an unexpected result. CRPS-1 is more common in females than males, 3–4:1 (our study 70:14), as are most types of chronic pain; however, most of the genetic results’ power came from our male cohort.^40 41^ It will need further studies to determine if there is an altered sex ratio effect for a CRPS-1 genetic background, to determine if there are more SNPs to be identified, and if our results are relevant to other ethnic groups. Murine studies have shown a sex difference in chronic pain after injury, with T-cells being important rather than macrophages in females; whereas macrophages and microglia are important in males, but not T-cells.^42 43^ This raises the possibility of different mechanisms of disease in males and females in CRPS-1 and that therapeutic responses may also be influenced by sex. Experimentally this would suggest that attention should be given to the choice of appropriate XX or XY cell lines, and choice of sex and age of experimental animals.

We acknowledge that our genetic results may be only part of the CRPS-1 story; autoimmune disease is more common in women than men, and recent studies strongly suggest it could be causative in some cases of CRPS-1.^44^ IgG from patients with late stage CRPS caused pain hypersensitivity when injected into the hind-paw of injured mice, and this was associated with nociceptor sensitisation.^45–47^ Interestingly, the contralateral, uninjured paws retained normal sensitivity suggesting that CRPS-associated circulating pro-algesic IgG antibodies bind to targets only expressed after injury; alternatively IgG-bound functional elements such as receptors might become relevant in the context of co-factors released in the context of injury. It would be interesting to speculate that the degree of activation of regional immune cells after injury could be important for this process, but more research is needed to understand such mechanisms. In addition, agonist autoantibodies active against the sympathetic autonomic nervous system proteins ADRB1, ADRB2 and CHRM2 has been found in approximately one-third of CRPS cases.^48–50^ CRPS-1 could turn out to be a number of different diseases with the same phenotype, or at least the phenotype could be potentially stratified by SNPs and by sex. And both the autoantibodies and SNP rare alleles could target the same biological pathway.

There are several weaknesses in the design of the study which bear mention. The sample sizes are relatively small, which may well have precluded other SNPs from being detected. The design of the study to include a prospective replication cohort mitigates the risk of over-reporting the overexpressed genes. There is little functional work performed on these genes and their widespread distribution may well affect their biological role. In addition, it should be noted that this study included only Caucasians. CRPS-1 in Asian populations has different demographics, and likely different genetic drivers, for example, P2RX7 SNP allele frequencies differ significantly from those of Caucasians.^1 51 52^

Our data support an underlying genetic predisposition to CRPS-1 in up to a third of cases, with this effect being most prominent in males. The rare alleles of the SNPs we report here could lead to the discovery of CRPS-1-specific dysregulated cell lineage functions. Further studies are needed to confirm and expand our results to allow future precision diagnosis and personalised treatments for CRPS-1.

## Data Availability

Data are available upon reasonable request.
