# Supplementary material for: Evidence of a genetic background predisposing to complex regional pain syndrome type 1
Source: J Med Genet. 2023 Oct 10;61(2):163–70. doi: 10.1136/jmg-2023-109236 (PMC10850724; doi:10.1136/jmg-2023-109236)
Supplement: Supplementary data [file jmg-2023-109236supp001.pdf]

Supplementary Data Legends

Supplementary Table 1: Genetic results for the CRPS-1 Discovery Cohort

**Gene and SNP**	**SNP alleles**	**CRPS Discovery Cohort allele frequency (*n*=34)**	**1000Genomes allele Frequency (UK data) (*n*=100)**	**1000Genomes Chi Square *p* value (FDR corrected)**	**EVS allele frequency (European Americans) (*n*=4300)**	**EVS Chi Square *p* value (FDR corrected)**
**ANO10 rs41289586**	p.Arg263His	0.088 (6/68)	0.0096	1.52x10-6	0.026	0.018
**P2RX7 rs28360457**	p.Arg307Gln	0.059 (4/68)	0.0037	1.47x10-6	0.014	0.04
**PRKAG1 rs1126930**	p.Thr57Ser	0.103 (7/68)	0.009	7.5x10-7	0.034	0.05
**SLC12A9 rs80308281**	p.Ile350Thr	0.044 (3/68)	0.0005	6.91x10-11	0.003	8.53x10-5

Legend for Supplementary Table 1:

The SNP names with the gene they occur in, and common and rare alleles are shown in the first two columns. "*n*" is the number of individuals in the cohort. The study results are given for each SNP for the Discovery CRPS-1 Cohort as allele frequency uppermost, and then in brackets are the number of rare alleles over total alleles. Published SNP allele frequencies are given for 1000 Genome GBR cohort (most like our cohorts), and Exome Variant Server (EVS) European American cohort (predominantly northern European). For each SNP in the CRPS-1 Discovery Cohort, the statistical difference of a false discovery rate (FDR) corrected (against the total number of SNPs, see text) Chi-squared *p* value is given. This is shown against 1000 Genome reference data and EVS data separately.

Shaikh SS, *et al. J Med Genet* 2024; 61:163–170. doi: 10.1136/jmg-2023-109236

Supplementary Table 2: Genetic results for the CRPS-1 Replication Cohort

Gene and SNP	SNP alleles	CRPS Replication Cohort allele frequency (*n*=50)	1000 Genomes Allele frequency (UK data) (*n*=100)	Chi Square *p* value using 1000 Genomes	EVS Allele frequency (European Americans) *n*=4300	Chi Square *p* value using EVS
**ANO10 rs41289586**	p.Arg263His	0.05 (5/100)	0.0096	0.043	0.026	0.011
**P2RX7 rs28360457**	p.Arg307Gln	0.01 (1/98)	0.0037	0.55	0.014	1.0
**PRKAG1 rs1126930**	p.Thr57Ser	0.04 (4/100)	0.009	0.11	0.034	0.24
**SLC12A9 rs80308281**	p.Ile350Thr	0.02 (2/510)	0.0005	0.26	0.003	0.040

Legend for Supplementary Table 2:

The SNP names with the gene they occur in, and common and rare alleles are shown in the first two columns. "*n*" is the number of individuals in the cohort. The study results are given for each SNP for the Replication CRPS-1 Cohort as allele frequency uppermost, and then in brackets are the number of rare alleles over total alleles. Published SNP allele frequencies are given for 1000 Genome GBR cohort (most like our cohorts), and Exome Variant Server (EVS) European American cohort (predominantly northern European). For each SNP the statistical difference between the Replication Cohort and either 1000 Genomes or EVS was calculated using Chi-squared without Yates correction with two tailed to calculate the *p* value This is shown against 1000 Genome reference data and EVS data separately.

Shaikh SS, *et al. J Med Genet* 2024; 61:163–170. doi: 10.1136/jmg-2023-109236

Supplementary Table 3: Genetic results for the Chronic Pain Cohort

Gene And SNP	SNP alleles	Chronic pain cohort Allele frequency (*n*=39)	1000 Genomes Allele Frequency (UK data) (*n*=100)	Chi Square *p* value using 1000 Genomes	EVS Allele frequency (European Americans) *n*=4300	Chi Square *p* value using EVS
**ANO10** **rs41289586**	p.Arg263His	0 (0/78)	0.0096	1.0	0.026	0.628
**P2RX7** **rs28360457**	p.Arg307Gln	0 (0/78)	0.0037	1.0	0.014	0.628
**PRKAG1** **rs1126930**	p.Thr57Ser	0 (0/78)	0.009	1.0	0.034	0.644
**SLC12A9** **rs80308281**	p.Ile350Thr	0 (0/78)	0.0005	1.0	0.003	1.0

Legend for Supplementary Table 3.

The SNP names with the gene they occur in, and common and rare alleles are shown in the first two columns. The study results are given for each SNP in the Chronic Pain Cohort (CPC) as allele frequency uppermost, and then in brackets are the number of rare alleles over total alleles. Published SNP allele frequencies are given for 1000 Genome GBR cohort (most like our cohorts), and Exome Variant Server (EVS) European American cohort (predominantly northern European). For each SNP, the statistical difference between the Chronic Pain Cohort (CPC) and either 1000 Genomes or EVS was calculated using Chi-squared without Yates correction two tailed to calculate the *p* value This is shown against 1000 Genome reference data and EVS data separately.

Shaikh SS, *et al. J Med Genet* 2024; 61:163–170. doi: 10.1136/jmg-2023-109236

Supplementary Table 4: Individual Discovery CPS-1 cohort genetic results of exome analysis versus Sanger sequencing

**Gene** / **SNP** / **Patient Identifier**	ANO10 rs41289586	P2X7 rs28360457	PRKAG1 rs1126930	SLC12A9 rs80308281
	Exome/Sanger	Exome/Sanger	Exome/Sanger	Exome/Sanger
1	CC/CC	GG/GG	GG/GG	TT/TT
2	CC/CC	GG/GG	GG/GG	TT/TT
3	**CT/CT**	GG/GG	GG/GG	TT/TT
4	CC/CC	**GA/GA**	**GC/GC**	**TC/TC**
5	**CT/CT**	GG/GG	GG/GG	TT/TT
6	**CT/CT**	GG/GG	GG/GG	TT/TT
7	CC/CC	GG/GG	GG/GG	TT/TT
8	**CT/CT**	**GA/GA**	GG/GG	TT/TT
9	CC/CC	GG/GG	GG/GG	TT/TT
10	CC/CC	GG/GG	GG/GG	TT/TT
11	CC/CC	GG/GG	GG/GG	TT/TT
12	CC/CC	GG/GG	GG/GG	**TC/TC**
13	CC/CC	GG/GG	**GC/GC**	TT/TT
14	CC/CC	GG/GG	GG/GG	TT/TT
15	CC/CC	GG/GG	GG/GG	TT/TT
16	CC/CC	GG/GG	GG/GG	TT/TT
17	CC/CC	**GA/GA**	GG/GG	TT/TT
18	CC/CC	GG/GG	**GC/GC**	TT/TT
19	CC/CC	GG/GG	GG/GG	TT/TT
20	CC/CC	GG/GG	GG/GG	TT/TT
21	CC/CC	GG/GG	GG/GG	TT/TT
22	CC/CC	GG/GG	GG/GG	TT/TT
23	CC/CC	GG/GG	GG/GG	TT/TT
24	CC/CC	GG/GG	GG/GG	TT/TT
25	CC/CC	**GA/GA**	GG/GG	**TC/TC**
26	**CT/CT**	GG/GG	GG/GG	TT/TT
27	CC/CC	GG/GG	GG/GG	TT/TT
28	CC/CC	GG/GG	GG/GG	TT/TT
29	**CT/CT**	GG/GG	GG/GG	TT/TT
30	CC/CC	GG/GG	GG/GG	TT/TT
31	CC/CC	GG/GG	**CC/CC**	TT/TT
32	CC/CC	GG/GG	GG/GG	TT/TT
33	CC/CC	GG/GG	**CC/CC**	TT/TT
34	CC/CC	GG/GG	GG/GG	TT/TT

Legend for Supplementary Table 4.

The patient Identifier is the chronological order that they entered the study. Genes and their study SNP head columns. For each person and for each SNP the genotyped alleles are shown. The genotype from exome analysis is shown first, then the genotype from Sanger sequencing. Results which deviate from homozygous wild type are bolded. Each exome result was

initially checked by use of the Integrated Genome viewer (IGV) using the bam and bam.bai files of each individual's data. Sanger sequencing was then performed using the original genomic DNA sample donated by each individual. There were no ambiguous exome or Sanger sequencing results.

Supplementary Table 5: Genetic results for the study cohorts

**Gene And SNP**	**SNP alleles**	**CRPS Discovery Cohort allele frequency (*n*=34)**	**1000 Genomes Allele Frequency (UK data) (*n*=100)**	**1000 Genomes Chi Square *p* value (FDR corrected)**	**EVS Allele frequency (European American) (*n*=4300)**	**EVS Chi Square *p* value (FDR corrected)**	**CRPS Discovery and Replication Cohorts allele frequency (*n*=84)**	**CRPS combined cohort data uncorrected *p* value against EVS**	**UK biobank Allele frequency (*n*=488,377)**	**CRPS combined cohort data uncorrected *p* value against UK biobank**	**Chronic Pain Cohort Allele frequency (*n*=39)**
**ANO10 rs41289586**	p.Arg263His	**0.088 (6/68)**	**0.0096**	1.52x10-6	**0.026**	0.018	**0.065 (11/168)**	0.0016	**0.0277**	0.0029	**0 (0/39)**
**P2RX7 rs28360457**	p.Arg307Gln	**0.059 (4/68)**	**0.0037**	1.47x10-6	**0.014**	0.04	**0.030 (5/166)**	0.0869	**0.0177**	0.236	**0 (0/39)**
**PRKAG1 rs1126930**	p.Thr57Ser	**0.103 (7/68)**	**0.009**	7.5x10-7	**0.034**	0.05	**0.054 (11/168)**	0.0267	**0.0336**	0.0221	**0 (0/39)**
**SLC12A9 rs80308281**	p.Ile350Thr	**0.044 (3/68)**	**0.0005**	6.91x10-11	**0.003**	8.53x10-5	**0.029 (5/168)**	0.0001	**0.0049**	0.0001	**0 (0/39)**

Legend for Supplementary Table 5:

The SNP names with the gene they occur in, and common and rare alleles are shown in the first two columns. In each cohort or population database title, "*n*" refers to number of people in a cohort/study/database. The study results are given for each SNP for the discovery CRPS-1 Discovery Cohort and combined CRPS-1 cohorts, and the Chronic Pain Cohort (CPC). Published SNP allele frequencies are given for 1000 Genome GBR cohort (most like our cohorts), the Exome Variant Server (EVS) European American cohort (predominantly northern European), and the UK biobank. Cohort study results are given for each SNP as allele frequency uppermost, and then in brackets are the number of rare alleles over total alleles. For each SNP in the CRPS-1 Discovery Cohort, the statistical difference of a false discover rate (fdr) corrected (against the total number of SNPs, see text) Chi-squared without Yate's correction two tailed *p* value is given. This is shown against 1000 Genome reference data and EVS data separately. For the combined CRPS-1 cohort (Discovery and Replication cohort) results we conducted a Chi-squared (without Yates correction) two tailed test against EVS and UK biobank SNP allele frequencies. The final column shows the results of an fSNPd discovery analysis for our Chronic Pain Cohort (CPC) with all four SNPs with genotypes and coverage confirmed; statistics are not given as no individuals of the cohort had any of the CRPS-1 SNP rare alleles.

Shaikh SS, *et al. J Med Genet* 2024; 61:163–170. doi: 10.1136/jmg-2023-109236

BMJ Publishing Group Limited (BMJ) disclaims all liability and responsibility arising from any reliance placed on this supplemental material which has been supplied by the author(s)

Shaikh SS, *et al. J Med Genet* 2024; 61:163–170. doi: 10.1136/jmg-2023-109236
